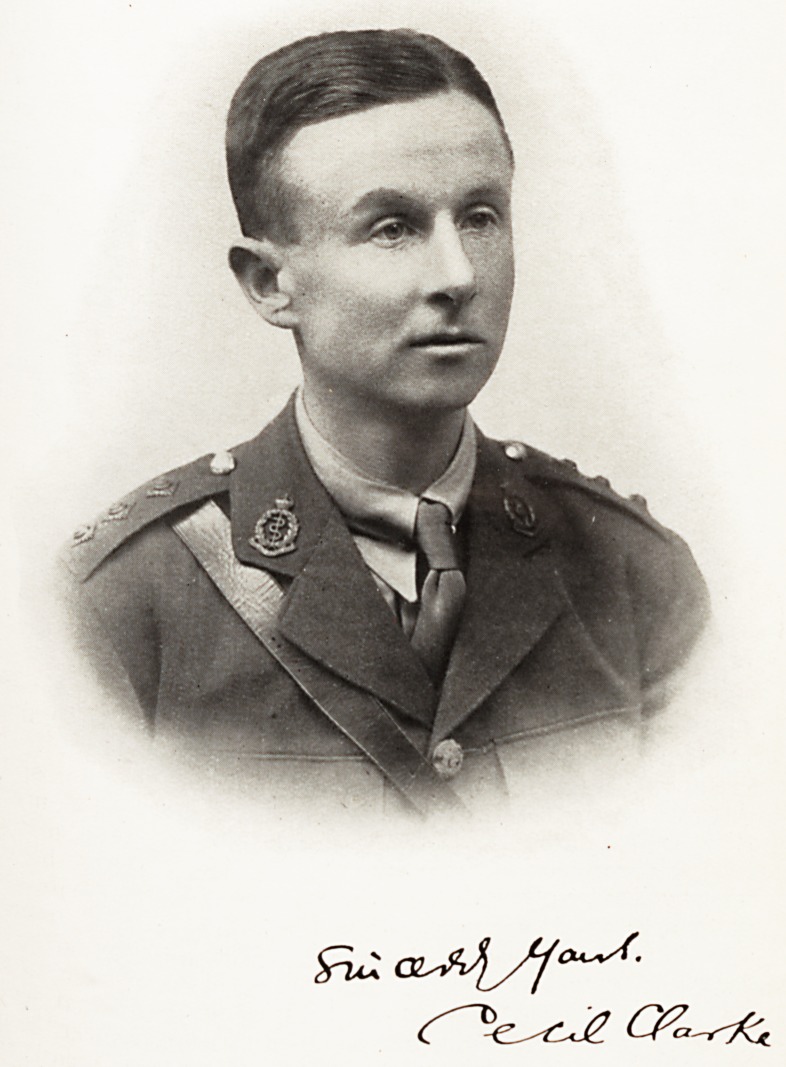# Cecil Clarke

**Published:** 1925-01

**Authors:** 


### CECIL CLARKE, M.D., B.S., M.R.C.P. Lond.

Cecil Clarke was born in Bristol in 1885, the son of Mr. Leslie M. Clarke. After an education at the Warminster Grammar School he joined the Bristol Medical School. His clinical appointments were done at the General Hospital. There he gained the Henry Marshall Prize, the Clarke Surgical Scholarship, and the Martyn Memorial Scholarship in 1908, and the Sanders Scholarship in 1909. In 1908 he became qualified, receiving both the M.B., B.S. degrees of the University of London and the diploma of the Conjoint Board. In 1908 he was appointed Assistant House Physician and in 1909 House Surgeon to the Hospital. After these, and a voyage to the Far East and back, he went to the Brompton Hospital, where he was successively Assistant Resident Medical Officer, House Physician, and Assistant in the Pathological Department; this last after an illness—acute pleurisy following a skating accident and a long drive in wet clothes—which was the first experience of the broken health which was destined to trouble him for the rest of his life. His next step, after a short rest, was to return to London for postgraduate work at University College (where he gained much from the teaching of Sir John Rose

Sincerely Yours.

Cecil Clarke

Bradford) and at the London Hospital. Then he joined a British Red Cross Unit which served with the Bulgarian Army in the Second Balkan War, a service of hardship, exposure and privation, in which he so distinguished himself as to gain several British and Bulgarian decorations.

After taking his M.R.C.P. Lond. in 1913 he went to Germany, first to Berlin and later to Dresden, where he spent a long time in the Pathological Department under Professor Schmorl. Thence he went on to Vienna, where he had been but a few days when war broke out. He went back to Dresden at once, and was interned there for several months. His knowledge of German enabled him to escape, though he was nearly caught. As soon as he got home, without leaving London, indeed, he volunteered for overseas service, and after a short interval received a lieutenant's commission in the R.A.M.C., but was sent to a laboratory in Belgium, where he did excellent work in the typhoid fever endemic among the civil population, and was of great value in helping to turn this danger aside from the armies in the field. It was at this time that he was selected for professional service to a certain exalted personage, an incident of which he was proud, though he never spoke of it till after the war, and then only to one or two people. Thence he was transferred to Salonika, where he worked with Professor Dudgeon, carrying out some valuable researches into the morbid histology of malaria that was subsequently published (see list of papers). In Macedonia he became infected with dysentery, but with characteristic grit he stuck to his job till 1917, when he was invalided home. He used his sick leave to get some of his research work written up for publication, and as soon as he was fit he went abroad again, back to France, first with a C.C.S., and subsequently with a Battalion of the Sherwood Foresters at his own request. At the Armistice he was recalled for bacteriological work at an officers' hospital in Llangammarch Wells, and in 1919, on demobilisation, he came back to Bristol to take up the Medical Registrarship at the General Hospital. He also acted as Pathologist to the Hospital in an interregnum, and was next year appointed Assistant Physician. He was also appointed Physician to the Queen Victoria Jubilee Convalescent Home.

In 1922 his health was so poor that he went into a sanatorium at Ipswich, and in that same year his appendix, the site of a chronic abscess, was removed at Guy's Hospital. This, he hoped, would get rid of the cause of his ill-health, but it was soon clear that the old lung infection was progressing, though very slowly. Doubtless it had been lit up by the dysentery which he acquired in Macedonia. However, he put up a most courageous fight, in which he could never be got to admit defeat; indeed, only a few weeks before his death he was talking of the work he would do when he came back to the Hospital. At the end of 1924 he went to Switzerland for treatment, but became worse, and died three weeks later, on the last Sunday of the year, at Montana.

In October, 1919, he married Miss Nora Martin, who nursed him with untiring devotion throughout his long illness.

A memorial service held at St. Paul's Church, Clifton, on January 2nd, 1925, was attended by many of his colleagues, who will always hold their memory of Clarke in affectionate esteem.

This record of a short but crowded life gives but a bare idea of the indomitable spirit of the man, a heroic spirit which made the most of those high qualities inherent in him even in the teeth of such a succession of illnesses as we have recorded. We add two high appreciations from intimate friends who worked with him. One (a member of a London hospital staff) says: "He was a good fellow, and to my mind exceptionally able and possessed of a first-rate critical faculty. I learned to admire his character in France, where, in his unassuming way, his laboratory work was only rivalled by his courage in insisting on serving with a Battalion, when his poor health and special attainments would have secured for him important laboratory work. On the eve of an attack near Ypres the poor fellow developed a painful ischio-rectal abscess; but rather than leave the line, he got his corporal to open it with a penknife in the trench. The same courage carried him through these last few years with little or no lament, but I know how disappointed he continually was at being kept from the work he loved."

Another, speaking of his work in Bristol after the war, says: "He was a purist in his use of English. Redundancy, slipshod grammar, vague expression, gave him almost physical pain. He was widely read in the English classics, and intimately acquainted with scientific German and French. He was a keen but just critic with an uncanny gift for fastening on the truth and rejecting what was unproven or unsound. To these gifts he had added an exceptionally wide experience of pathology and medicine, and in his neat, well-ordered mind had evolved a clear and comprehensive view of the work to which he hoped to devote his energies. To him pathology was always the foundation of medicine; medicine without pathology was unsound, and pathology without medicine fruitless."

Had he lived, Clarke would doubtless have achieved fame both for himself and for his School. His zest for work and passion for truth must have won for his natural gifts some notable triumph in the struggle against disease. Yet it may be doubted whether, even so, he could have achieved any triumph so splendid as that assertion of the supremacy of the spirit over the body of which his life was so brilliant an example. In support of this contention may be read an article by Clarke himself on his experiences as a patient in *The Stethoscope* for October, 1922. His other published papers were :—

"Report on a Series of Relapses in an Epidemic of Enteric Fever," *J. Roy. Army Med. Corps*, 1915.

"Search for Typhoid Carriers among 800 Convalescents" (jointly), *Lancet*, 1916.

"Cultivation of the Malarial Parasite" (jointly), *Ibid.*, 1917.

"Investigation on Fatal Cases of Pernicious Malaria caused by Plasmodium Falciparum in Macedonia" (jointly), *Quar. J. Med.*, 1919.

"Congenital Disease of Pancreas with Infantilism" (jointly), *Ibid.*, 1924.

"Renal Efficiency," *Bristol M.-Chir. J.*, 1922.

## Figures and Tables

**Figure f1:**